# Poly (ADP-ribose) polymerase 1 mRNA levels strongly correlate with the prognosis of myelodysplastic syndromes

**DOI:** 10.1038/bcj.2016.127

**Published:** 2017-02-17

**Authors:** P Diamantopoulos, K Zervakis, P Zervakis, M Sofotasiou, T Vassilakopoulos, I Kotsianidis, A Symeonidis, V Pappa, A Galanopoulos, E Solomou, E Kodandreopoulou, V Papadopoulou, P Korkolopoulou, M Mantzourani, G Kyriakakis, N-A Viniou

**Affiliations:** 1 Hematology Unit, First Department of Internal Medicine, Laikon General Hospital, National and Kapodistrian University of Athens, Athens, Greece; 2 Department of Hematology, University Hospital of Alexandroupolis, Alexandroupoli , Greece; 3 Department of Internal Medicine, University Hospital of Patras, Rio, Greece; 4 Haematology Division, Second Department of Internal Medicine, Attikon General Hospital, National and Kapodistrian University of Athens, Athens, Greece; 5 Department of Clinical Hematology, 'G. Gennimatas' District General Hospital, Athens, Greece; 6 Department of Pathology, National and Kapodistrian University of Athens, Athens, Greece

## Abstract

Poly (ADP-ribose) polymerase 1 (PARP-1) has a central role in the repair of DNA breaks and is a promising treatment target in malignancy. We measured PARP1 mRNA levels by a SYBR-green-based PCR in the bone marrow of 74 patients with myelodysplastic syndrome (MDS) and correlated them to their demographic, hematologic and prognostic characteristics. The median PARP1 mRNA levels were correlated to the type of MDS (2008/2016 WHO classification, *P*=0.005) and to the IPSS score (*P*=0.002). A correlation was also found with the IPSS-R score (*P*=0.011) and the cytogenetic risk (*P*=0.008). In all cases, higher PARP1 levels were correlated with a higher risk category. Moreover, we found a significant survival disadvantage for patients with high PARP1 levels (median survival of 37.4 months versus ‘not reached’ for low PARP1 levels, *P*=0.0001, and a 5-year survival rate of 29.8 versus 88.9%, respectively). PARP1 mRNA levels were found to be the stronger predictor of survival in multivariate analysis. These correlations have never been reported in the past and may render PARP1 a prognostic factor to be incorporated in the current prognostic systems for MDS, also laying the basis for clinical trials evaluating PARP1 inhibitors in higher-risk MDS.

## Introduction

The Poly (ADP-ribose) polymerases (PARPs) comprise a family of nuclear enzymes that upon binding to DNA breaks, polymerize, and by poly (ADP-ribosylation), participate in DNA repair and gene transcription.^1, 2^

Among the members of the PARP family, PARP1 is the most abundant. It has an important role in the repair of single-strand DNA (ssDNA) and double-strand DNA (dsDNA) breaks. Inhibition of PARP1 activity leads to reduced DNA break repair, eventually resulting to cell death. PARP1 has a low enzymatic activity, which is stimulated by allosteric activators, such as damaged DNA (single- and double-strand breaks, crossovers, cruciforms and supercoils), undamaged DNA structures, nucleosomes and some protein-binding partners. Binding of PARP1 with such molecules boosts its enzymatic activity that targets core histones, histone H1 and transcription-related factors,^3, 4, 5, 6, 7^ and recruits various proteins involved in the DNA damage response to the sites of DNA damage,^3^ acting as a DNA damage sensor.^4^ Although low levels of DNA damage trigger its detection and repair, high levels of DNA damage may lead to cell death by either apoptosis or necrosis through PARP1 over-activation,^8^ which causes depletion of the cellular NAD+ and ATP pool.^9, 10^ In general, actively proliferating cells (such as malignant cells) are more sensitive to PARP1 activation and die by necrosis, while non-proliferating cells are resistant to cell death under the same conditions.^11, 12^ Moreover, PARP1 is an upstream molecule of autophagy,^13^ a cellular degradation process, utilized by cancer cells as a survival mechanism to overcome stresses. When stress reaches a critical point, autophagy has been hypothesized to mediate cell death.^14^

It has been shown that PARP1 is not a necessary molecule under normal conditions, since mice with a silenced *PARP1* gene are healthy, although they bear genetic instability. However, when DNA damage is higher than the intrinsic levels, exceeding the levels at which the repair enzymes can function, PARP1 becomes important for the cell response to DNA damage.^15^

The pharmaceutical research focuses on the development of potent competitive inhibitors of PARP1/NAD+.^16, 17^ The use of PARP1 inhibitors mainly aims to the sensitization of malignant cells to cytotoxic agents, thus leading to treatment potentiation. PARP1 inhibition leads to ‘preservation’ of DNA damage that would have otherwise been repaired by the base excision repair (BER) system and to dysfunction of the malignant cell, although there is evidence that BER dysfunction may explain one aspect of the propensity to chromosomal breaks in some patients with MDS.^18^

Due to the multiple roles of PARP1, studies about the role of PARP1 in certain hematologic malignancies have conflicting results. However, PARP1 overexpression has been correlated with poor treatment response in children with acute lymphoblastic leukemia,^19^ while PARP1-driven apoptosis has been shown to be important in patients with chronic lymphocytic leukemia.^20^ Moreover, PARP1 inhibitors have been tested *in vitro* in hematologic malignancies, mostly lymphoid malignancies,^21, 22^ but also in AML, MDS and acute promyelocytic leukemia (APL).

In the present study, we investigate the role of PARP1 in patients with MDS by measuring PARP1 mRNA and protein levels and correlating them with the type of MDS according to the 2008^23^ and 2016^24^ World Health Organization (WHO) classification of MDS and with the risk for AML transformation as well as the overall survival (OS) of the patients. Our aim was to investigate a potential prognostic role of PARP1 in MDS and possibly to identify patients that could benefit from treatment with PARP1 inhibitors.

## Patients and methods

### Patients

The study included patients diagnosed with MDS according to the 2008 WHO classification. Patients that would have been classified as having MDS based on the French–American–British (FAB) classification (that is, Chronic Myelomonocytic Leukemia (CMML) and Refractory Anemia with Excess Blasts in transformation (RAEB-t)) were excluded from the study.

We retrospectively recorded the demographic, clinical and hematologic characteristics of the patients that were included in the study. The patients were classified according to the 2008 and 2016 WHO classification of MDS, and the International Prognostic Scoring System (IPSS),^25^ the revised IPSS (IPSS-R)^26^ and the WHO Classification-Based Prognosis Scoring System (WPSS)^27^ for MDS.

## Methods

Bone marrow samples from all patients were collected in ethylenediaminetetraacetic acid (EDTA) during a routine bone marrow aspiration. All samples were processed within 6 hours from collection. Following RNA extraction and cDNA synthesis, the samples were kept at -80 °C. To measure PARP1 mRNA levels, we used a quantitative real-time polymerase chain reaction (qRT-PCR).

### RNA extraction and reverse transcription

The Trizol protocol (Invitrogen, Carlsbad, CA, USA) was used to extract and purify total RNA from bone marrow samples. Reverse transcription was performed using an MMLV-derived reverse transcriptase enzyme (M-MLV RT, Invitrogen), according to standard protocols.

### Primer design for Real-Time PCR

Primers for PARP1 and β-actin were designed with the help of the primer3 software (University of Massachusetts, USA), using the relevant annotated cDNA sequences from NCBI BLAST (NM_001618.3 for PARP1 and NM_001101.3 for β-actin). Primer sequences: for PARP1 forward, 5′-CCTGATCCCCCACGACTTT-3′ reverse, 5′-GCAGGTTGTCAAGCATTTC-3′ and for β-actin forward, 5′-AGGATGCAGAAGGAGATCACT-3′ reverse 5′-GGGTGTAACGCAACTAAGTCATAG-3′.

### Real-time PCR

Real-time PCR was performed with the use of 2X iTaq Universal SYBR GREEN Supermix (Bio-Rad Laboratories, Hercules, CA, USA) on a CFX96 Real-time PCR system (Bio-Rad Laboratories) using the following cycling conditions for both PARP1 and β-actin: 5′′ at 95 °C, 15′′ at 59 °C and 5′′ at 72 ºC, all steps repeated for 40 cycles. Relative quantitation of PARP1 and β-actin transcripts was performed with the standard curve method. PARP1 mRNA levels were expressed as a ratio of PARP1/actin transcript levels.

### Immunoblotting

Total cellular protein was obtained from each sample, using RIPA buffer. Lysates were incubated on ice for 10 min and then centrifuged for 10 min at 14 000 rpm. Protein extracts were then separated by SDS-PAGE electrophoresis on acrylamide 4% stacking and 8% separating gels, using the Mini-Protean electrophoresis cell (Bio-Rad Laboratories), per standard procedures. Proteins were transferred from the gel to a PVDF membrane (Immun-blot PVDF, Bio-Rad Laboratories), per the manufacturer’s instructions. Membranes were then incubated in a blocking solution for 1 h at room temperature and the primary antibody was added at a dilution 1/1000 - PARP rabbit mAb, No. 9542 or β-actin rabbit polyclonal Ab, #4967 (Cell Signaling Technology, Danvers, MA, USA) after reprobing the membranes for loading control. After an overnight incubation at 4 °C, the membrane was washed in TBS-T and incubated with the secondary antibody at a dilution 1/1000 in a blocking buffer for 1 h at room temperature (anti-rabbit IgG, HRP conjugated, #7074, Cell Signaling Technology). After 3 × washes in TBS-T, the signal was detected with ECL Blotting reagent (Clarity Western ECL Substrate, #170-5061, Bio-Rad Laboratories). Detection of PARP1 bands was performed using simple films (RX1318 Fuji - X-ray film SuperRX), and the Azure c300 Chemiluminescent Western Blot Imaging System (Azure Biosystems, Dublin, CA, USA).

### Statistical analysis

IBM SPSS statistics, version 23.0 (IBM Corporation, North Castle, NY, USA) was used for the statistical analysis of the results. The individual tests used are cited in the ‘Results’ section, separately for each correlation.

## Results

**Figure 1:**
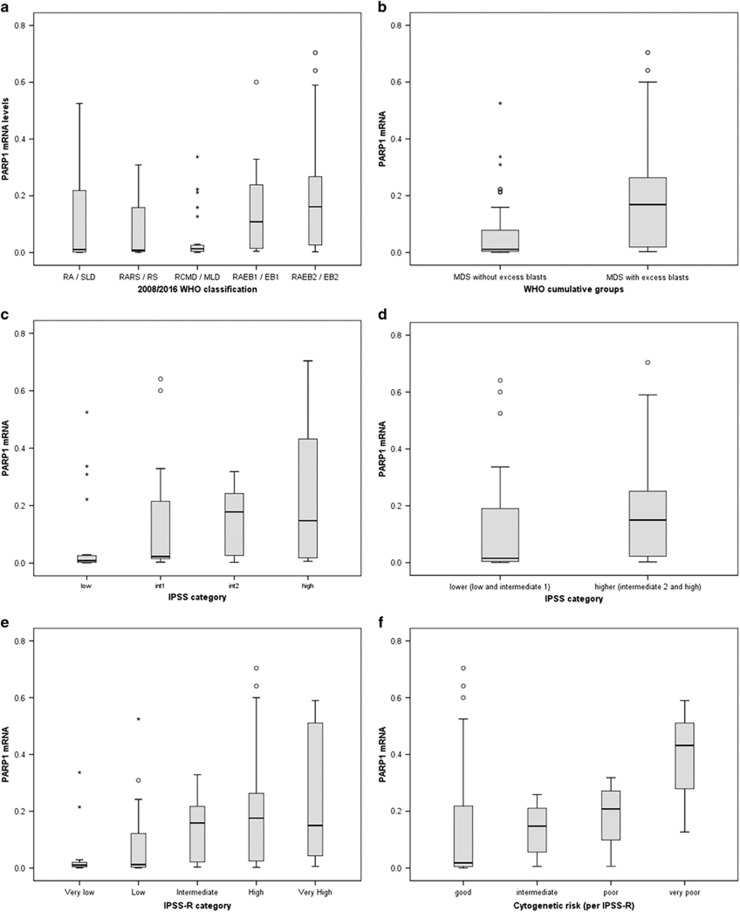
Box plots for the distribution of PARP1 mRNA levels (**a**) in the different types of MDS according to the 2008/2016 WHO classification, (**b**) in the WHO cumulative groups (MDS without excess blasts and MDS with excess blasts), (**c**) in the risk groups according to IPSS, (**d**) in the cumulative risk groups according to IPSS (lower, incorporating low and intermediate-1, and higher, incorporating intermediate-2 and high), (**e**) in the risk groups according to IPSS-R, and (**f**) in the cytogenetic risk groups (per IPSS-R). IPSS, international prognostic scoring system; IPSS-R, revised international prognostic scoring system; MDS, myelodysplastic syndrome; MLD, multilineage dysplasia; RA, refractory anemia; RARS, refractory anemia with ring sideroblasts; RCMD, refractory cytopenia with multilineage dysplasia; RS, ring sideroblasts; SLD, single lineage dysplasia; RAEB, refractory anemia with excess blasts; EB, excess blasts; WHO, world health organization.

Seventy-four (74) patients with MDS were included in the study. The baseline demographic and hematologic characteristics of the patients are shown in Table 1. The clear majority of the patients were treatment naive, since only 5 (6.7%) had been treated with a hypomethylating agent before sample collection. The median PARP1 mRNA levels, expressed as a ratio of PARP1/actin transcript level, were 0.0264 (range 0.0003–3.4040). We found that the PARP1 mRNA levels showed a statistically significant correlation to the type of MDS, according to the 2008 and 2016 WHO classification (Independent Samples Kruskal–Wallis Test, two-sided *P*=0.005 for both classifications, detailed results in Table 1, box plot in Figure 1a). In both WHO classifications, patients with MDS without excess blasts had significantly lower levels of PARP1 mRNA as shown in Figure 1b (Independent Samples Mann–Whitney *U* Test, two-sided *P*=0.0001).

The levels of PARP1 mRNA were also found to be correlated to the IPSS score of the patients (Independent Samples Kruskal–Wallis Test, two-sided *P*=0.002, detailed results in Table 1). The corresponding box plot is shown in Figure 1c. Patients with lower (low and intermediate-1)-risk MDS had almost 10 times lower PARP1 mRNA levels than patients with higher (intermediate-2 and high)-risk MDS (0.0155 versus 0.1500), and although some overlap exists, the result was highly significant (Independent Samples Mann–Whitney *U* Test, two-sided *P*=0.003; Figure 1d).

Comparisons were also carried out with the IPSS-R and WPPS scores, and the results showed a statistically significant difference in the levels of PARP1 mRNA among the several categories of IPSS-R (Independent Samples Kruskal–Wallis Test, two-sided *P*=0.011, Figure 1e) but not among the categories of WPSS (Independent Samples Kruskal–Wallis Test, two-sided *P*=0.111), although there was a trend for higher PARP1 mRNA levels in higher-risk categories. When merging very low, with low and intermediate scores of IPSS-R forming a ‘lower’ category and high with very high scores forming a ‘higher’ category as was done with IPSS, PARP1 mRNA levels were significantly lower in the ‘lower’ category (Independent Samples Mann–Whitney *U* Test, two-sided *P*=0.004). A correlation of the PARP1 mRNA levels with the cytogenetic risk (per IPSS-R categorization) was also found (Independent Samples Median test *P*=0.008), as shown in Figure 1f.

**Figure 2:**
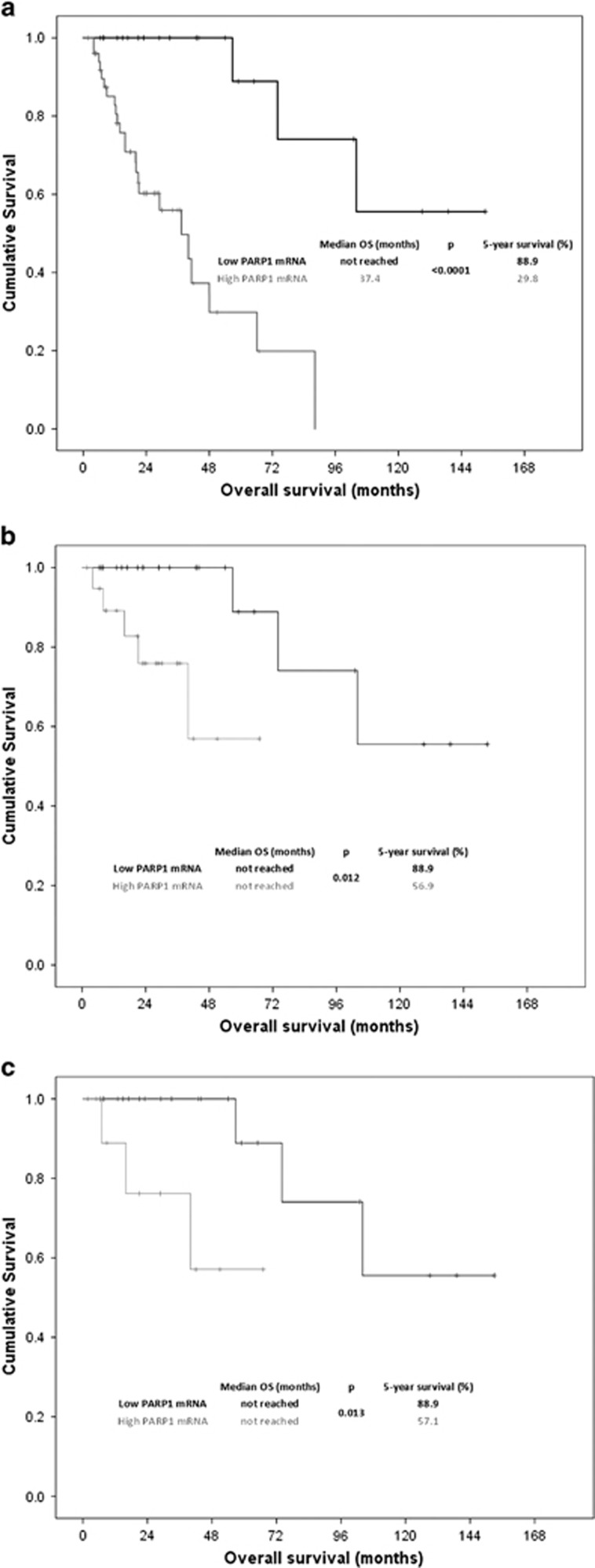
Overall survival in relation to PARP1 mRNA levels; OS was evaluated at the cutoff of 0.011, which was estimated to be the best cutoff level of the present series. Results for the whole cohort (**a**), for the subgroup of patients without excess blasts per the 2016 WHO classification (**b**), and for the subgroup of patients with lower risk (low and intermediate-1) per IPSS (**c**). OS, overall survival; PARP1, poly [ADP-ribose] polymerase 1.

The median OS of the cohort was 66.1 months. The median OS of patients with high PARP1 mRNA levels was much shorter than that of patients with low PARP1 mRNA levels, as shown in Figure 2a (using a cutoff point for PARP1 mRNA levels of 0.011, median survival was not reached versus 37.4 months in the low and high PARP1 groups, respectively, log rank *P*=0.0001). The 5-year survival rate of patients with high PARP1 levels was 29.8 versus 88.9% for those with low levels. PARP1 levels could also discriminate patients with lower OS from patients with higher OS, when tested in the lower (low and intermediate-1) IPSS subgroup (log rank *P*=0.012, Figure 2b). The same applied for patients with MDS without excess blasts, according to the 2008 and 2016 WHO classification (log rank *P*=0.013, Figure 2c). PARP1 levels could not further discriminate patients with lower OS from patients with higher OS in the rest of the subgroups tested (that is, higher IPSS, and MDS with excess per the 2016 WHO classification).

Univariate cox regression analysis showed that PARP1 mRNA levels had an impact on OS (hazard ratio 13.75, *P*=0.001). The hazard ratio for PARP1 mRNA levels was higher than any of the variables tested. WHO classification, IPSS, IPSS-R, WPSS, percentage of bone marrow blasts, cytogenetic risk and cytopenias had an impact on OS, while sex, age, hemoglobin level, peripheral blood neutrophil and platelet count did not.

Cox regression survival analysis was performed for PARP1 levels in comparison to WHO categories, IPSS and its components (percentage of bone marrow blasts, cytogenetic risk and cytopenias) and IPSS-R. PARP1 was found to have a greater impact on survival than any of the other tested variables (hazard ratio 6.53–12.06), as shown in detail in Table 2.

Immunoblotting for PARP1 was performed in all samples. The protein could not be detected in any of the patient samples with the simple x-ray film, but it was detected in all (11) samples that were tested with the Azure c300 Chemiluminescent Western Blot Imaging System. However, we did not proceed to the analysis of all samples for financial reasons. Thus, the results presented here constitute just an approximation of the expression of the protein in patients with MDS and could not be used for further statistical analysis. The median value of PARP1 expression (detected with the Azure c300 system) was 0.298 (0–1.625) and no correlations with the WHO classification or the IPSS were detected. To our knowledge, PARP1 detection by western blotting has never been performed in the past in samples of patients with MDS.

## Discussion

It has been shown *in vitro* that PARP1 has an important role in the pathways of apoptosis and necrosis. Cell death studies lead to the implication of PARP1 in the process through two opposite ways. First, suppression of PARP1 activity leads to failure of DNA break repair, cell function derangement and consequently cell death. On the other hand, PAPR1 inhibitors were shown to have significant efficacy in the treatment of diabetes, inflammation, septic shock and neuron death in experimental models. These beneficial effects were attributed to the prevention of the consequences of PARP1 over-activation under cytotoxic stress conditions, leading to cell lysis.

The correlation of higher levels of PARP1 mRNA with higher (intermediate-2 and high) IPSS risk MDS has never been reported so far. The result remains valid when using the IPSS-R as well. This correlation is biologically reasonable, since higher-risk MDS bear a larger accumulation of DNA breaks that induces PARP1 overexpression. The absence of a statistically significant correlation with the categories of WPSS (although there was a trend for higher levels of PARP1 in higher WPSS groups) may be partly explained because WPSS also includes transfusion dependence, a factor that is not considered for the calculation of IPSS and IPSS-R. Transfusion dependence is a clinical parameter with potentially minor correlation to DNA damage. Since PARP1 is a molecule directly correlated with DNA damage, this lack of correlation seems reasonable. The strong correlation of higher levels of PARP1 with higher cytogenetic risk (per both IPSS and IPSS-R) is another significant and reasonable correlation that underscores the potent role of PARP1 in the pathophysiology of MDS. Finally, there was a strong prognostic significance of PARP1 mRNA levels for the OS of patients with MDS, as shown by univariate and multivariate Cox regression analysis. Moreover, PARP1 was also important as a prognostic factor in patients with lower IPSS because higher PARP1 levels were correlated with lower OS in this subgroup of patients. The same did not apply in the higher IPSS subgroup, probably because in this group of patients, the low OS has been already defined by the high cytogenetic risk and bone marrow blasts. This finding is very significant, since lower-risk patients could be divided in subcategories based on PARP1 levels, and those with higher PARP1 levels could be managed more aggressively.

Nevertheless, the physiologic role of PARP1 overexpression in MDS has not been yet fully elucidated. Thus, the verification and further evaluation of this finding in larger patient series may have several important implications.

First, the correlation of PARP1 load with the WHO classification of MDS and the IPSS and IPSS-R scores, as well as with the OS may constitute a new prognostic factor for MDS that could be incorporated in the current prognostic systems. Quantifying PARP1 mRNA or protein levels in larger patient series may set a cutoff point that could help towards this direction. Moreover, the significantly lower levels of PARP1 in patients with MDS without excess blasts in comparison with those with MDS with excess blasts indicates that distinct pathophysiologic processes may govern these entities.

Secondly, this result can form the basis for the design of phase I clinical trials evaluating the use of PARP1 inhibitors in patients with higher-risk MDS. At least five PARP inhibitors have been used in clinical trials for the treatment of solid tumors. The initial reports suggest very little toxicity of these agents, a fact that is very important for the frail and elderly patients with MDS. However, PAPR inhibitors have not been tested in clinical trials for hematologic malignancies, although there have been several reports of their *in vitro* efficacy in both lymphoid and myeloid malignancies, alone or in combination with other agents.

Olaparib, an orally bioavailable PARP inhibitor, has been shown to cause synergistic lethality in base excision repair-deficient cells that are treated with a combination of decitabine and olaparib,^28^ suggesting that combination treatment of hypomethylating agents with PARP inhibitors might improve outcomes of patients with MDS or AML. The same agent has been shown to induce death in 88% of primary AML case samples and cell lines, sparing normal lymphocytes and without substantially affecting normal bone marrow CD34-positive cells.^29^ Finally, it has been shown that PARP1 inhibitors may have a synergistic effect in the apoptosis of APL cells when these cells are treated with a combination of all-*trans* retinoic acid (ATRA) and PARP1 inhibitors.^30^

PARP1 is also related to DNA methylation, a stable epigenetic signal that can be perpetuated postmitotically through the clone and is correlated to gene expression silencing. PARP1 may affect methylation through the regulation of the expression of the DNA methyltransferase-1 (*DNMT1*) gene, or through direct regulation of the protein activity. PARP1 binds to the gene promoter and protects it from methylation, thus inducing *DNMT1* activation. Loss of the expression of DNMT1 causes hypomethylation of the whole genome.^31^ This correlation of PARP1 with methylation is very important, since hypermethylation is a major event in the pathogenesis of MDS, and hypomethylating agents are the most effective available treatment option in patients with MDS. The synergistic effect with decitabine, described above, is promising evidence that PARP1 inhibitors could be used in patients with MDS in combination with hypomethylating agents such as decitabine or 5-azacytidine that constitute the standard of care for MDS.

## Figures and Tables

**Table 1:** Patients’ characteristics and results

*Characteristic*		*Result*		
Number of patients, *N* (%)		74 (100)		
Sex (Male to female ratio)		1.61		
Age (years), median (range)		74.5 (32–91)		
Previous treatment, *N* (%)^a^		5 (6.7)		
*MDS type (WHO classification)*, N *(%)*			*PARP1 mRNA, median (range)*	P
*2008*	*2016*			0.005^b^
RA	MDS-SLD	7 (9.5)	0.0108 (0.0010–0.5250)	
RARS	MDS-RS	3 (4.1)	0.0086 (0.0026–0.3089)	
RCMD	MDS-MLD	25 (33.8)	0.0137 (0.0007–0.3370)	
RAEB-1	MDS-EB1	16 (21.6)	0.1086 (0.0047–0.6002)	
RAEB-2	MDS-EB2	23 (31.1)	0.1733 (0.0026–3.4040)	
*MDS (based on WHO classification)*, N *(%)*				0.000^c^
Without excess blasts (RA, RARS, RCMD)		35 (47.3)	0.0110 (0.0003–0.5250)	
With excess blasts (RAEB-1, RAEB-2)		39 (52.7)	0.1688 (0.0026–3.4040)	
*IPSS*, N *(%)*				0.002^b^
Low		22 (29.7)	0.0087 (0.0003–0.5250)	
Intermediate 1		21 (28.4)	0.0229 (0.0036–0.6410)	
Intermediate 2		18 (24.3)	0.1784 (0.0026–1.0900)	
High		13 (17.6)	0.1477 (0.0060–3.4040)	
*IPSS*, N *(%)*				0.003^c^
Lower (low and intermediate 1)		43 (58.1)	0.0155 (0.0003–0.6410)	
Higher (Intermediate 2 and high)		31 (41.9)	0.1500 (0.0026–3.4040)	
*IPSS-R*, N *(%)*				0.011^b^
Very low		11	0.0107 (0.0010–0.3370)	
Low		16	0.0123 (0.0007–0.5254)	
Intermediate		11	0.1589 (0.0036–0.3290)	
High		24	0.1759 (0.0026–1.0900)	
Very high		7	0.1500 (0.0060–3.4040)	
*WPSS*, N *(%)*				0.111^b^
Very low		8	0.0067 (0.0014–0.3089)	
Low		19	0.0108 (0.0007–0.5250)	
Intermediate		9	0.0185 (0.0003–0.6002)	
High		25	0.1733 (0.0026–3.4000)	
Very high		9	0.1873 (0.0060–0.4316)	
*Cytogenetic risk (per IPSS-R)*, N *(%)*				0.008^b^
Good		51	0.0182 (0.0003–1.0900)	
Intermediate		11	0.1477 (0.0060–0.2590)	
Poor		4	0.2082 (0.0062–0.3184)	
Very poor		3	0.4316 (0.1270–0.5901)	

Abbreviations: IPSS, international prognostic scoring system; IPSS-R, revised international prognostic scoring system; MDS, myelodysplastic syndrome; MLD, multilineage dysplasia; PARP1, poly [ADP-ribose] polymerase 1; RA, refractory anemia; RAEB, refractory anemia with excess blasts; EB, excess blasts; RARS, refractory anemia with ring sideroblasts; RCMD, refractory cytopenia with multilineage dysplasia; RS, ring sideroblasts; SLD, single lineage dysplasia; WHO, world health organization; WPSS, WHO Classification-Based Prognosis Scoring System.

a Treatment with a hypomethylating agent.

b Independent Samples Kruskal–Wallis Test, two-sided *P*.

c Independent Samples Mann–Whitney *U* Test, two-sided *P*.

**Table 2:** Multivariate analysis of overall survival

	*Multivariate Cox regression analysis*		
	*HR*	*95% CI*	P*-value*
*Model A-OS*			
PARP1	6.74	1.08–42.25	0.040
WHO (with or without excess blasts)	2.38	0.70–8.13	0.173
			
*Model B-OS*			
PARP1	6.53	1.20–35.58	0.030
IPSS (lower versus higher)	2.68	0.99–7.24	0.053
			
*Model C-OS*			
PARP1	7.64	1.40–41.80	0.019
IPSS-R (lower versus higher)	1.66	0.60–4.58	0.331
			
*Model D-OS*			
PARP1	10.84	1.31–89.52	0.027
Bone marrow blasts	3.65	0.75–17.76	0.110
Cytopenias	0.25	0.09–0.68	0.007
Cytogenetic risk	3.65	0.75–17.76	0.009

Abbreviations: IPSS, International Prognostic Scoring System; OS, overall survival; PARP1, poly [ADP-ribose] polymerase 1; R, Revised; WHO, World Health Organization.

Model A-OS concomitantly assessed PARP1 mRNA levels at the cutoff of 0.011 and WHO classification (MDS with excess blasts versus MDS without excess blasts). Model B-OS concomitantly assessed PARP1 mRNA levels at the cutoff of 0.011 and IPSS (lower (low and intermediate-1) versus higher (intermediate-2 and high) score). Model C-OS concomitantly assessed PARP1 mRNA levels at the cutoff of 0.011 and IPSS-R (lower (very low, low and intermediate) versus higher (high and very high) score). Model D-OS concomitantly assessed PARP1 at the cutoff of 0.011 with the individual components of IPSS (bone marrow blasts - <5%, 5–20%, cytogenetic risk – low, intermediate, high, and number of cytopenias – <1 and >1).
